# Bibliometric analysis of scientific production in occupational health nursing

**DOI:** 10.47626/1679-4435-2023-1135

**Published:** 2024-02-16

**Authors:** Miguel Valencia-Contrera, Flérida Rivera-Rojas

**Affiliations:** 1 Universidad Andrés Bello, Facultad de Enfermería, Santiago, Chile; 2 Universidad Católica del Maule, Departamento de Enfermería, Curicó, Chile

**Keywords:** bibliometrics, occupational health nursing, occupational health, nursing research, nursing, bibliometría, enfermería del trabajo, salud ocupacional, investigación en enfermería, enfermería

## Abstract

Occupational health nursing, formerly known as industrial nursing, which in turn has its foundations in public health, develops preventive, assistance, legal, and expert activities, as well as management, teaching, and research, the last of which updates knowledge and provides answers to questions arising from clinical experience. The aim of this article was to analyze the scientific production about occupational health nursing in Scopus and Web of Science databases. This is a retrospective, descriptive, bibliometric study on the scientific production about occupational health nursing in which scientific evidence was analyzed and characterized using Bibliometrix software. An unequal scientific production was evidenced, with a higher proportion of publications in countries such as the United States, South Korea, Brazil, and Spain; furthermore, there has been a sustained growth in the number of publications, whose topics of greatest interest were associated with health, risks, exposures, and care.

## Abstract (es)

La enfermería de salud laboral, antiguamente denominada enfermería industrial, que a su vez presenta sus cimientos en la salud pública, desarrolla actividades preventivas, asistenciales, legales y periciales, gestión, docencia e investigación, esta última, actualiza el conocimiento y da respuesta a preguntas que nacen de la experiencia clínica. El objetivo del presente artículo fue analizar la producción científica en enfermería del trabajo en las bases de datos Scopus y Web of Science. Se trata de un estudio bibliométrico, retrospectivo y descriptivo sobre la producción científica de enfermería del trabajo; para ello, se analizó y caracterizó la evidencia científica, a través del software Bibliometrix. Se evidenció una producción científica desigual, con una mayor representación en países como Estados Unidos, Corea del Sur, Brasil y España; adernás, se identificó un crecimiento sostenido en el número de publicaciones, cuyos tópicos de mayor interés han sido asociados a la salud, los riesgos, exposiciones y el cuidado.

## INTRODUCTION

Occupational health nursing had an ancient beginning, whose foundations have been described since 1878, when it was named industrial nursing, and Philippa Flowerday is considered the first nurse of this specialty. At that time, nursing care was focused on sick workers in their household, and thus care was focused on their family in a holistic manner^1^; industrial nursing, in turn, originated from public health, being therefore more focused on protection.^2^

In 1915, nurses in the area of Boston organized the first industrial nurses' association,^3^ which responded to the need of an optimal organization for the development of labor activities, merging the theory-practice-methodology triad. However, this activity has not been recognized as a specialty in some countries, such as Chile.^4^

Occupational health nurses develop strategies and actions to prevent accidents in employees' place of work or diseases secondary to labor activities; moreover, they encourage learning of self-care practices that improve workers' lifestyle and enable to achieve biopsychosocial stability in work teams.^5^ Considering that work may have a positive or negative effect on workers, there is evidence that occupational health professionals are capable of identifying health determinants and pursue workers' well-being, for which these workers should be involved in the development of healthy behaviors, thus favoring productivity of companies and reducing costs.^6^

In order to support the occupation health nursing practice, some investigations have been developed to provide scientific evidence, update knowledge, and give answer to questions arising from clinical experience,^7^ which makes it possible to consolidate specific knowledge, providing support to specific clinical practices in occupational safety and health.^8^ In view of the foregoing, the present study was conducted aiming to analyze scientific production on occupational health nursing in Scopus and Web of Science (WoS) databases.

## METHODS

This is a descriptive, retrospective, bibliometric study on the scientific production about occupational health nursing in which scientific evidence was analyzed and characterized through the Bibliometrix software. The Scopus and WoS databases were searched using the "Occupational Health Nursing" descriptor, validated in the DeCS thesaurus, and defined as the nursing practice in the workplace,^9^ it is important to note that the descriptor used, "occupational health nursing" in English, has the following synonyms: "industrial nursing," "industrial nursings," and "occupational health nursings." Data collection was conducted on July 10, 2022.

## RESULTS

### WEB OF SCIENCE DATABASE

**Figure 1.**
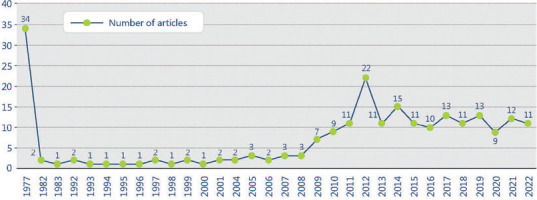
Number of articles organized per year of publication in Web of Science.

Search on the WoS database using the search strategy "all fields" identified 232 publications, which were published from 1977 to July 2022 (period when the search was conducted). There was an average of 3.19 citations by publication. Figure 1 shows the number of articles identified per year.

In turn, documents were obtained from 61 sources of information (journals, books, etc.), with *Workplace Health & Safety* journal standing out as the source with the greatest number of articles, accounting for a total of 60, followed by *Occupational Health Nursing* journal, with 33 articles, and *Acta Paulista de Enfermagem* journal, with 20 articles. Table 1 presents the first 20 journals in descending order.

**Table 1.** Number of articles by journal in Web of Science

Journal	Number of articles
*Workplace Health Saf*	60
*Occup Health Nurs*	33
*Acta Paul Enferm*	20
*AAOHN J*	19
*Rev Lat Am Enfermagem*	9
*J Adv Nurs*	8
*Rev Esc Enferm USP*	6
*J Occup Health*	4
*Occup Med (Lond)*	4
*Enferm Clin*	3
*Int Nurs Rev*	3
*J Korean Acad Nurs*	3
*Med Pracy.*	3
*Public Health Nurs*	3
*Rev Fun Care Online*	3
*Am J Ind Med*	2
*Int J Environ Res Public Health*	2
*J Dermatol Nurses Assoc*	2
*Rev Rene*	2
*Rev Enferm*	2

**Figure 2.**
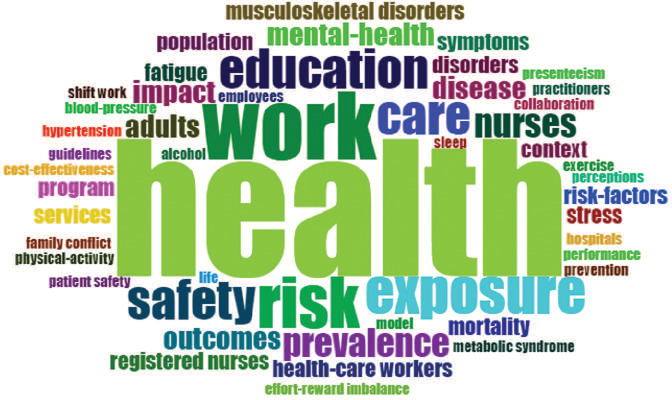
Word cloud of the most used terms.

With regard to the terms used in the sample analyzed, "health" stands out the most used one, followed by "work" "risk," "exposure," "care," "education," and "safety". The 50 most used terms were selected to generate a word cloud, with words sized in proportion the number of occurrences (Figure 2).

In relation to country of origin of publications, 24 countries were identified, with the United States, Brazil, and Spain leading the scientific production in the area, which may be explained by existing collaboration networks between these countries.

### SCOPUS DATABASE

**Figure 3.**
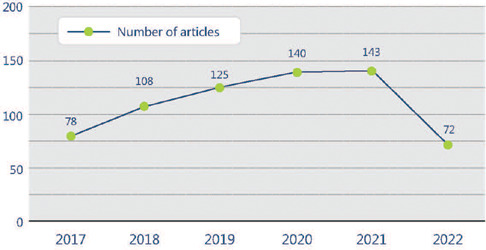
Number of articles organized by year of publication in Scopus.

Search on the Scopus database using the search strategy "all fields" identified a total of 6,750 studies published from 1945 to July 2022 (period when the search was conducted). The scientific production published in the last 5 years (2017-2022) were analyzed, remaining a sample of *666* documents, with an average of 3.377 citations per publication. Figure 3 shows the number of published articles per year.

In turn, 261 sources of information were identified, of which *Workplace Health & Safety* journal stands out again as the source with the greatest number of articles, accounting for 99, followed by the *Journal of Korean Academy Of Nursing Administration* journal, with 63 articles, and by the *Journal of Korean Academy of Community Health Nursing* journal, with 20 articles. Table 2 presents the first 20 journals in descending order.

**Table 2.** Number of articles organized by journal in Scopus

Journal	Number of articles
*Workplace Health Saf*	99
*J Korean Acad Nurs Adm*	63
*J Korean Acad Community Health Nurs*	31
*J Korean Acad Nurs*	20
*Korean J Adult Nurs*	15
*Indian J Public Health Res Dev*	13
*J Nurs Manag*	13
*Int J Environ Res Public Health*	12
*J. Korean Fund. Nurs*	12
*J Adv Nurs*	10
*Asia Life Sci*	9
*J Clin Nurs*	9
*J Occup Health*	7
*Enferm Clin*	5
*J Korean Acad Soc Nurs Edu*	5
*Nurs Open*	5
*Sangyo Eiseigaku Zasshi*	5
*Arch. Mai Prof. Environ.*	4
*Child Health Nurs Res*	4
*Ind Health*	4

**Figure 4.**
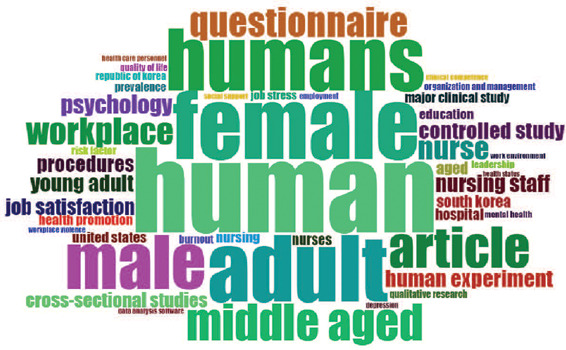
Word cloud of the most used terms.

With regard to the terms used in the sample analyzed, "human" stands out as the most used one, followed by "female," "adult," "humans," "male," and "occupational health nursing". The first 50 terms more used were selected to generate a word cloud, with words sized in proportion to the number of occurrences (Figure 4).

In relation to country of origin of publications, 53 countries were identified, with South Korea, the United States, China, and Brazil leading the scientific production in the area.

## DISCUSSION

The scientific evidence analyzed shows that occupational health nursing has an emerging development, since, although the WoS database has presented a fluctuating behavior over the last 10 years, an ascending pattern was identified in the Scopus database, with 78 publications in 2017 and 143 in 2021, and to date, at the beginning of the second semester of 2022, 72 publications were identified, and thus this pattern is thus expected to continue.

It is worth noting the role played by *Workplace Health & Safety* journal, which is the official publication of the American Association of Occupational Health Nursing (AAOHN), since it is the source of most results identified. It is worth clarifying the evolution of the journal, since it has presented four names through its history; initially, *American Association of Industrial Nurses Journal,* from 1953 to 1968^10^; subsequently, *Occupational Health Nursing,* from 1969 to 1985^11^; a few times later, *AAOHN Journal: Official Journal of The American Association of Occupational Health* Nurses, from 1986 to 2011^12^; and finally, *Workplace Health & Safety,* from 2012 onwards.^13^ Therefore, the official journal of the AAOHN was found to be the first, second, and fourth leading journal with the highest number of publications identified in the WoS database.

The journal with the third highest number of publications was *Acta Paulista de Enfermagem,* which was also the Latin American journal with the highest number of publications. In relation to the Scopus database, there was a significant number of journals from Eastern Asia, specifically from Korea, accounting for the second, third, fourth and fifth highest number of publications in this database.

These results may be explained by collaborations described since 2007,^14^ which were created with the purpose of approaching the global crisis of scarcity and unequal distribution of nurses, including members of Australia, Hong Kong, Japan, South Korea, Philippines, Thailand, the United Kingdon, and the United States.

The scientific representation of the aforementioned countries is consistent with the development of specific training that they achieved, in line with the finding shown in an analysis of skills of training programs in occupational health nursing, which observed that professionals had graduate training in occupational risks presented greater scientific production than those who did not have this training.^15^ This finding enables to explain the limited development of research in this area in countries that do not presented the specialty in occupational safety and health.^16^ such as Chile,^4^ revealing the importance of focusing efforts on the development of training programs.

The situation described above gains special importance when it is pointed out that occupational health nursing has progressed in parallel to developments in occupational health^2^ and to an improvement in the quality of care provided,^17^ because its field of competence includes preventive, assistance, legal, and expert activities, as well as management, teaching, and research.^18^

With regard to the limitations of the present study, they lie on the descriptor used, because it may not properly reflect the production on occupational health nursing in its multiple lines of action, but rather considers those articles that used this descriptor. Furthermore, only two databases were analyzed; although they were those with the greatest impact, they do not incorporate the most emerging journals, thus excluding them from the analysis. Conversely, in relation to its strengths, the present article is the first manuscript, to authors' knowledge, which analyzed the scientific production in the area, which allows for evidencing the extent of scientific development in occupational health nursing, as well exposing spaces of opportunity to further develop this specialty.

## FINAL CONSIDERATIONS

The present article provided answers to the proposed goal of analyzing the scientific production on occupational health nursing, evidencing an unequal scientific production, with a higher proportion of publications in countries such as the United States Unidos, South Korea, Brazil, and Spain; furthermore, there has been a sustained growth in the number of publications, whose topics of greatest interest were associated with health, risks, exposures, and care.
